# M1M2: Deep-Learning-Based Real-Time Emotion Recognition from Neural Activity

**DOI:** 10.3390/s22218467

**Published:** 2022-11-03

**Authors:** Sumya Akter, Rumman Ahmed Prodhan, Tanmoy Sarkar Pias, David Eisenberg, Jorge Fresneda Fernandez

**Affiliations:** 1 Martin Tuchman School of Management, New Jersey Institute of Technology, Newark, NJ 07102, USA; 2 Department of Computer Science, Virginia Tech, Blacksburg, VA 24061, USA; 3 Department of Information Systems, Ying Wu College of Computing, New Jersey Institute of Technology, Newark, NJ 07102, USA

**Keywords:** emotion recognition, CNN, EEG, machine learning, sensor, deep learning

## Abstract

Emotion recognition, or the ability of computers to interpret people’s emotional states, is a very active research area with vast applications to improve people’s lives. However, most image-based emotion recognition techniques are flawed, as humans can intentionally hide their emotions by changing facial expressions. Consequently, brain signals are being used to detect human emotions with improved accuracy, but most proposed systems demonstrate poor performance as EEG signals are difficult to classify using standard machine learning and deep learning techniques. This paper proposes two convolutional neural network (CNN) models (M1: heavily parameterized CNN model and M2: lightly parameterized CNN model) coupled with elegant feature extraction methods for effective recognition. In this study, the most popular EEG benchmark dataset, the DEAP, is utilized with two of its labels, valence, and arousal, for binary classification. We use Fast Fourier Transformation to extract the frequency domain features, convolutional layers for deep features, and complementary features to represent the dataset. The M1 and M2 CNN models achieve nearly perfect accuracy of 99.89% and 99.22%, respectively, which outperform every previous state-of-the-art model. We empirically demonstrate that the M2 model requires only 2 seconds of EEG signal for 99.22% accuracy, and it can achieve over 96% accuracy with only 125 milliseconds of EEG data for valence classification. Moreover, the proposed M2 model achieves 96.8% accuracy on valence using only 10% of the training dataset, demonstrating our proposed system’s effectiveness. Documented implementation codes for every experiment are published for reproducibility.

## 1. Introduction

Emotion is a complex, subjective experience characterized by physiological arousal, expressive behaviors, and cognitive processes. In many cases, emotions are triggered by specific events or stimuli and can be positive (e.g., joy, love, hope) or negative (e.g., anger, fear, sadness). Playing a vital role in people’s physical and mental health, emotions can influence one’s thoughts, feelings, and behavior.

Constantly processing information, the human brain uses emotions to make sense of its environment. However, we heavily rely on technology to communicate in an increasingly connected world. Emotion recognition is the ability of computer technology to identify and respond to human emotions. An electroencephalogram (EEG) [1,2] is a test that detects brain electrical activity using sensors (electrodes) attached to the scalp. EEG can be used to help diagnose conditions including seizures [3,4], sleep disorders [5,6], and head injuries [7], as well as for emotion recognition. This technology is still in its early stages, but it has the potential to transform how we communicate with each other and with machines, as well as improve our understanding of how the brain processes emotions.

The DEAP multimodal dataset [8] contains EEG, peripheral physiological, and audiovisual recordings from participants while they watched a series of videos for recognizing emotion. This dataset has four interconnected labels arousal, valence, dominance, and liking. The proposed model of this study uses the two labels of arousal and valence when conducting experiments using EEG data.

Numerous authors have researched emotion recognition from EEG data with different modeling, feature extraction, and preprocessing techniques [9,10]. However, a system’s performance is not only measured by accuracy. In addition to recognition accuracy, the performance of an optimal system can also consider such important measurements as robustness, execution time, learning with limited data, and learning with limited parameterized models. This paper’s study is therefore designed with such best practices in mind. The following specific research questions shape this paper:

1. Research question 1 (RQ1): Which deep learning model is more effective for EEG as a form of time series signal recognition?
2. Research question 2 (RQ2): Can feature extraction improve the recognition accuracy of a deep learning model? What is the most effective feature set?
3. Research question 3 (RQ3): What is the minimum EEG signal required for good recognition? Can it be done in real-time?
4. Research question 4 (RQ4): How much data and time is required for training the model to achieve a reasonable performance?

These four research questions form the basis of the research studies outlined in this paper. The DEAP dataset [8] has four labels: arousal, valence, dominance, and liking. Interestingly, only valence and arousal are responsible for basic emotions, such as happiness, sadness, surprise, disgust, anger, etc. [11] This study begins by sending raw EEG data from the DEAP dataset [8] through various deep learning models, including Long short-term memory (LSTM), Bidirectional LSTM (bi-LSTM), and CNN to discover which deep learning model performs best (RQ1). Through this methodology, the CNN model of this paper demonstrated the best performance when compared to LSTM and Bidirectional LSTM. For this reason, the CNN model is then selected and used for all subsequent experiments with different model architectures of 1D-CNN. Furthermore, by utilizing Fast Fourier Transform (FFT) for feature extraction, even better accuracy is attained (RQ2).

A total of five different 1D-CNN models are tested with residual connections to obtain state-of-the-art accuracy. Of these five models, two are selected for further experimentation. Of the two selected models, the first model, called M1, is heavy and consists of four convolution layers and three dense layers. M1 performs better than any other tested model. The second model, called M2, is lightweight and consists of two convolutional (Conv) layers and one dense layer. M2 is selected because it maintained the parameter data ratio. The remainder of the experiments is performed on this lightweight (M2) model (RQ1).

The methodology of this paper demonstrates better emotional recognition using minimal EEG data through varying window sizes on the lightweight (M2) model (RQ3). Subsequently, various combinations of train–test splits show that the lightweight (M2) model also performs well on a smaller amount of training data, even showing very good results on only fifty percent of training data (RQ4). Here, the model training time and testing time are also recorded to evaluate the real-time usability of the system (RQ4).

### Our Major Contributions

1. We propose a CNN model (M1) with residual connections, which achieved state-of-the-art accuracy (average accuracy: 99.89%) over the previous models.
2. We introduce a light version of the CNN model (M2) with only 7.69% parameters for the dataset size, which performs similarly (average accuracy: 99.22%) to the M1 model.
3. FFT is used for frequency domain features, convolutional layers for deep features, and complementary features to represent the dataset which proved to be very effective in emotion recognition from EEG.
4. Through experimentation, it is determined that only two seconds of EEG signal are required to achieve a good recognition (valence accuracy: 99.22%).
5. We empirically demonstrate that our model can achieve good accuracy (valence accuracy: 99.19%) by using only 50% of the dataset.

The rest of the paper is organized as follows: the literature review is organized by feature extraction methods and model techniques, providing an overview of previous studies and their limitations. The methodology section explains the dataset, preprocessing techniques, feature extraction method, data preparation method, and deep learning models. The experiment and results section demonstrates the experimental results based on the stated research questions. The future research and implications section exposes the limitations of this study and paves the way for future works, and then leads to the paper’s conclusion.

## 2. Literature Review

Research with neurophysiological sensors [12,13] has begun to aid in monitoring schizophrenia [14,15], Parkinson’s’ disease [16], traumatic brain injury [17], physiological signals [18], cognitive function [19], epileptic seizures [20], alcoholism [21], brain tumors [22], brain cancer [23], mental stability [24], personality [25], eye tracking [26], and many other phenomena. Moreover, studies conducted on EEG data processing have identified human emotions with exceptional accuracy [27,28,29,30,31,32,33,34,35,36,37,38,39,40]. These studies are the backbone of the growing number of emotional recognition applications, and because of their far-reaching applications, the societal impact of enhancing their efficiency, accuracy, and precision cannot be overstated.

While many emotion recognition studies have collected EEG data from human subjects, the process of doing so can be both time-consuming and expensive due to the highly sensitive equipment necessary to elicit EEG from participants. A popular alternative that helps expand the diversity of research possible is the creation and use of public datasets. Researchers generate these datasets and make them available for others to conduct further studies. Public datasets available for emotion recognition include the DEAP [8], SEED [41], LUMED [42], DREAMER [43], MAHNOB-HCI [44], and AMIGOS [45] datasets. Among these, the DEAP dataset is the most popular and widely used for emotion recognition from brain signals.

This study selected DEAP as the data source for EEG signals. The emotion labels in the DEAP include valence, arousal, dominance, and liking. As shown in Table 1, prior research with the DEAP has used a range of modeling frameworks, emotion labels, and different numbers of classifications.

### 2.1. Feature Extraction Methods

Generally, an important step for machine learning systems is to extract features from a training dataset. Various distinct methodologies have been applied for feature extraction of EEG data [1,2]. Among these methods, differential entropy (DE) [46,47], FFT [48], Wavelet-based features [58], Hjorth [49], Sequence Backward Selection (SBS) [49], Band power [50], power spectral density (PSD) [47,51], and sample entropy (SE) [52] are often employed to obtain good accuracy when working on valence, arousal, and other emotion labels from the DEAP dataset.

Some studies also include feature extraction techniques such as Deep Feature Clustering (DFC) [53], Higuchi’s fractal dimension (HFD) [54], Entropy [54], CNN feature extraction [59], Stats [55], raw [60], time frequency domain [56], Empirical Mode Decomposition (EMD) [57], Intrinsic Mode Functions Power (IMFP) [49], and Intrinsic Mode Functions Entropy (IMFE) [49].

Nevertheless, FFT [48] has been one of the most widely used methods for feature extraction in recent decades. In this study, FFT feature extraction is employed for extracting features from the DEAP dataset, as it has been proven in prior literature to be very effective for classifying an expansive number of emotions, including highly complex ones [48].

### 2.2. Machine and Deep Learning Modeling

Machine Learning (ML) statistics improves software performance by iteratively learning from training datasets, and identifying patterns in the data that were not specifically in the original computer program [61]. Closely related to statistical computation and mathematical optimization, ML applies to features learned from a training dataset [62], statistics such as classification or regression [63]. For this reason, ML traditionally requires a preprocessing step to extract features for the model to train on a training dataset.

Deep Learning (DL) is a specific subclass of machine learning, where the mode is built based on neural networks such as convolutional networks or long short-term memory networks. The DL models allow the building of complex predictive functions from the dataset. That is, an image of an entire person can be understood by the computer as corners or edges when using deep learning [63]. With its automated feature learning [64], DL models can achieve very good recognition accuracy training directly on raw datasets [62], rather than requiring a separate feature extraction step. This means that DL can automate the separation of discriminatory information from factors of variation for the learning task [63,64]. For example, to build an ML model for classifying cats and dogs, features such as height, weight, color, length, etc., need to be given as inputs to the model. However, those same images of cats and dogs could be taken directly as input when the DL model can automate the process of identifying their features. In the next section, numerous examples of these kinds of modeling techniques being used for emotion recognition from EEG signals with the DEAP dataset are evaluated.

#### 2.2.1. Deep Learning Approaches with DEAP

DL has been used to train on the DEAP dataset in numerous studies and has achieved notable recognition accuracy. Among the deep learning models, CNN and LSTM are most frequently used in prior research. Using the CNN model, Gao et al. [52] achieved 75.78% and 67.18% testing accuracy for arousal and valence, respectively. Their highest achieved accuracy for arousal and valence was 80.52% and 75.22%, respectively, using time and frequency features. Hasan et al. [48] used a 1D CNN with FFT in two and eight-class classifications. They achieved 96.63% and 96.17% for two-class classifications and 93.83% and 93.79% for eight-class classifications for valence and arousal. Alhalaseh et al. [54] used CNN along with naïve Bayes, k-nearest neighbor (k-NN), and decision tree (DT) for a two-class classification of emotion on valence and arousal from the DEAP dataset. They achieved their best accuracy of 95.20% with CNN. They used the Sample entropy (SE) and Higuchi’s fractal dimension (HFD) as feature extraction techniques.

Through a fusion of graph convolution neural networks and LSTM, Yin et al. [47] used differential entropy for extracting features to achieve 90.45% and 90.60% for valence and arousal, respectively. Garg et al. [55] used a merged LSTM as a modeling technique alongside wavelet-based features and statistical measures for extracting the features. They achieved 84.89%, 83.85%, 84.37%, and 80.72% for valence, arousal, dominance, and liking, respectively. In 2019, Ma et al. [60] used a multi-modal residual LSTM (MMResLSTM) with raw EEG, achieving 92.87% and 92.30% for arousal and valence, respectively. Anubhav et al. [50] used LSTM as well as K-nearest neighbor (KNN), support vector machine (SVM), decision tree, and random forest (RF) as classifiers. They achieved their best performance of 94.69% and 93.13% for arousal and valence, respectively, when they used LSTM with band power as a feature. Maeng et al. [51] proposed a genetic algorithm (GA) that used the LSTM deep learning model, where GA selected the effective parameters for LSTM. They used three domain sets for features and a generic algorithm for feature selection, achieving 91.3% and 94.8% for the valence and arousal labels, respectively. Kim et al. [59] combined LSTM, attention, and CNN for valence and arousal models with four-fold cross-validation. They achieved accuracies of 90.1% and 88.3% in the case of two-level classification, and 86.9% and 84.1% in the case of three-level classification.

Luo et al. [65] employed three algorithms to extract the EEG signals: wavelet-based features, variance, and FFT. The variance data processing method and spiking neural networks (SNN) was used to attain good accuracies for arousal, valence, dominance, and liking, which were 74%, 78%, 80%, and 86.27%, respectively. In 2019, Wang et al. [56] used Time-Frequency Domain EEG features on the binary classifier. Here, they experimented with three labels of the DEAP dataset, including arousal, valence, and dominance. They compared graph convolution neural networks (GCNN), SVM, and deep belief network (DBN) with their proposed phase-locking value (PLV) graph convolution neural networks (P-GCNN) model to achieve accuracy of 77.03%, 73.31%, and 79.20%, respectively. The DE-CNN-BiLSTM model, presented by Cui et al. [46], calculated differential entropy in several time slices of various frequency bands. They extracted the EEG signal’s highly complex attributes to produce 4D feature tensors based on their brain location. For the DEAP dataset, their method obtained an average accuracy of 94% for arousal and valence.

#### 2.2.2. Machine Learning Approaches with the DEAP

ML models have been alternatively more easily interpretable and lightweight [62]. Consequently, a number of research articles have used machine learning approaches to train on the DEAP. In 2021, Fang et al. [66] worked on two labels (arousal and valence) of the DEAP dataset with five classes of emotion (neutral, angry, sad, happy, and pleasant). They compared the results of their proposed Multi-Feature Deep Forest (MFDF) model with the different machine learning classifiers, including KNN, RF, and SVM. They used PSD and DE as feature extraction techniques to obtain an average accuracy rate of 71.05%, which was 3.40%, 8.54%, and 19.53% higher than RF, KNN, and SVM, respectively.

In 2020, Liu et al. [57] used an empirical mode decomposition (EMD) domain, combined with an optimal feature selection technique based on sequence backward selection (SBS) for feature extraction. They tested this method on two labels (valence and arousal) of the DEAP dataset. They then classified these labels with K-nearest neighbor (KNN) and support vector machine (SVM). With this methodology, they achieved an accuracy of 86.46% for valence and 84.90% for arousal.

In 2021, Cheng et al. [58] proposed a deep forest method for multi-channel EEG-based emotion recognition. Their approach could eliminate the necessity for feature extraction in traditional methods. They used binary class classification and attained an accuracy of 97.69% for valence and 97.53% for arousal on the DEAP dataset.

Shen et al. [67] employed the Multi-scale Frequency Bands Ensemble Learning (MSFBEL) modeling technique in 2021. These authors extracted features on two labels of the DEAP dataset by using differential entropy (DE) for four-class classification. They achieved an average accuracy of 74.22% under five-fold cross-validation.

In 2020, Asghar et al. [53] implemented deep feature clustering (DFC), rather than conventional feature selection techniques, to select high-quality characteristics. By eliminating useless characteristics, DFC reduces the time needed to train the network. To extract deep features, they employed four ready-trained deep neural networks (DNN). For SVM, KNN, and RF classifiers, respectively, they achieved 81.30%, 78.90%, and 80.70% accuracy, using the combined DNN model on valence and arousal labels of the DEAP dataset.

In 2021, Galvão et al. [49] proposed a model for predicting the exact values of valence and arousal in a subject-independent scenario. They used three Hjorth parameters (H1, H2, H3), spectral entropy, wavelet-based features, IMFP, and IMFE as feature extraction methods. They obtained their best results by experimenting with two models, KNN and RF. They classified the model into two and four classes and obtained an accuracy of 89.8% and 84.4%, respectively.

## 3. Methodology

In our case, we incorporate the feature extraction step into the DL model. FFT is used to extract frequency domain features, and then the extracted feature is fed into the CNN model for training, which proved better than using raw EEG.

**Figure 1:**
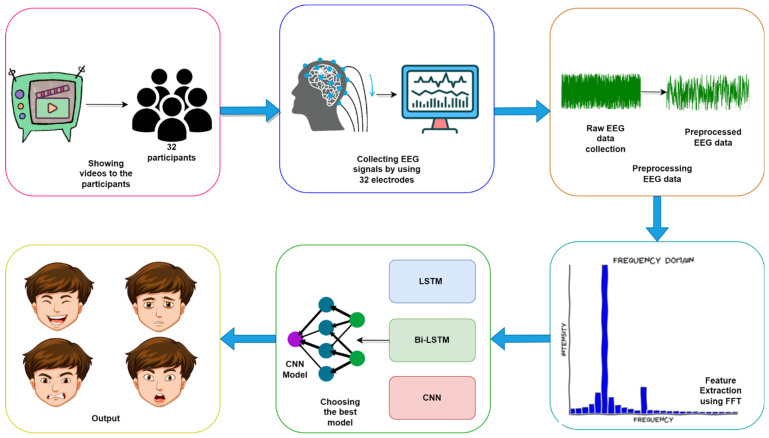
Complete workflow of the proposed emotion recognition pipeline from DEAP dataset.

Our study proposes an end-to-end EEG-based emotion recognition system. A complete workflow overview of the emotion recognition on the DEAP dataset is shown in Figure 1. Firstly, the DEAP dataset is selected as the source of raw EEG data. Then, the raw EEG data is pre-processed. To extract features from that preprocessed EEG data, FFT is used. Finally, the EEG data is passed into the model, and the model classifies the emotion from it.

### 3.1. The DEAP Dataset

**Figure 2:**
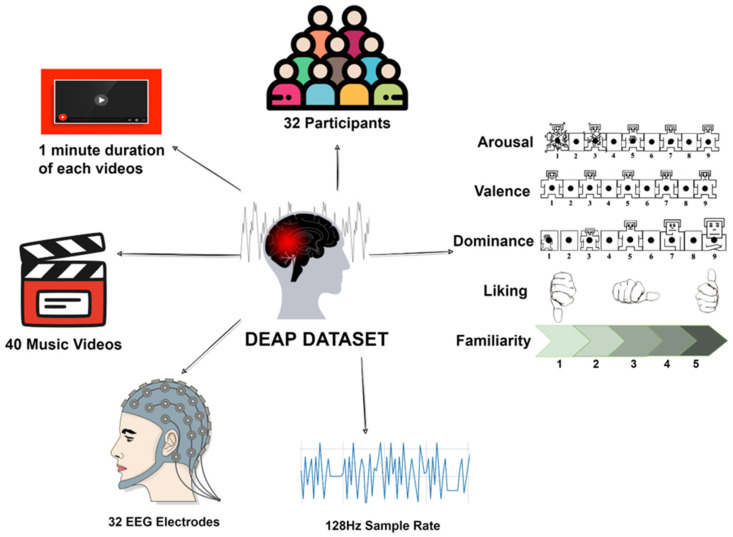
Overview of the DEAP dataset.

Koelstra et al. [8] created the freely accessible DEAP multi-modal dataset for emotion categorization, which includes physiological and EEG signals. It has a balanced male-to-female ratio and was created from the recordings of 32 participants, who ranged in age from 19 to 37. An overview of the DEAP is shown in Figure 2.

Of 120 music videos collected from the website last.fm [68] using affective tags and a manual process, 40 music videos were chosen, each one-minute long. A web-based subjective emotional assessment was used in choosing videos. Participants independently provided ratings of like/dislike, arousal, valence, and dominance. Among the 32 participants, 22 had frontal video of their faces recorded as well. Data was collected in Geneva for participants 23–32, while participants 1–22 were logged in Twente.

EEG was recorded from each participant with the use of 32 active AgCl electrodes, in a (.bdf) file captured with 48 channels at a 512 Hz sampling rate (placed according to the international 10–20 system [69]). EEG recordings were down-sampled to 128 Hz, and the blind signal separation was applied to it, which are available as two zip files in the DEAP.

**Figure 3:**
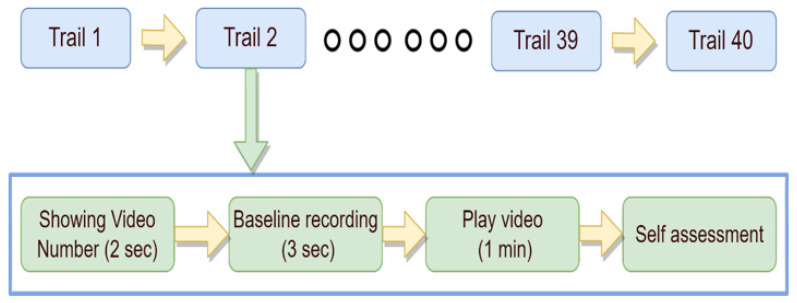
Experiment procedure of the DEAP for collection of EEG data.

The experimental procedure for collecting EEG data by the DEAP dataset team is shown in Figure 3. In order to synchronize EEG with emotion data, a fixation cross is first shown on the screen while each individual is instructed to relax for two minutes. Following that, each participant watches 40 one-minute videos in the trials. Prior to each trial, a two-second screen shows progress, and then a three-second fixation cross is shown to help the participant relax.

Galvanic Skin Response (GSR), an electrocardiogram, skin temperature, respiration amplitude, the blood volume measured by electromyograms of the Zygomaticus and Trapezius muscles, plethysmograph, and electrooculogram (EOG), among other peripheral physiological signals, are also recorded.

For each participant, the DEAP dataset provides two arrays shown in Table 2. One is a data array and another is a label array. In the data array, the array shape is 40 × 40 × 8064 and the contents are video/trial × channel × data. In the labels array, the array shape is 40 × 4 and the contents are video/trial × label (valence, arousal, dominance, liking). Each trial’s data size is 8064. This number comes from multiplying each recorded trial time (total 63 s: 60 one-second trials and a 3-s pre-trial baseline) and sample rate (128 Hz).

As there are a total of 32 participants, and each of them watches 40 trials, the total number of trials is 1280 (32 × 40). The total array size including every trial for the 32 participants of the DEAP dataset is shown in Table 2.

### 3.2. Preprocessing

EEG signals are naturally very noisy because many artifacts, such as eye movement, muscle movement, and head movement, are added when EEG data is recorded. Removing this noise, therefore, becomes a crucial step in the analysis. While raw data contains much noise and many artifacts, which can interfere with the analysis, the DEAP dataset team has actually provided a pre-processed version of their raw EEG data. This makes it free from any significant noise and easier to analyze.

This EEG data is downsized to 128 Hz from the original 512 Hz. The blind source separation technique is used to eliminate eye artifacts. The range of 4.0 Hz to 45.0 Hz band pass frequency filter is implemented. By following the commonly used references, the data is averaged. As the EEG data is recorded in two places, the Geneva order is followed for the EEG channels. A total of 63 s of trial data can be divided into each trial’s 60 s data and 3 s pre-trial baseline. Later, the 3 s of pre-trial baseline is removed. Finally, each video presentation is reordered to coincide with its experimental ID.

### 3.3. Feature Extraction

Machine learning models require features as inputs, while deep learning models can be trained on a raw dataset. Recent studies show that even deep learning models can achieve better recognition accuracy if meaningful features are extracted. Among feature extraction techniques, FFT has proven highly effective [70,71,72,73,74,75,76,77,78,79,80], including on the DEAP [48]. In this study, FFT was chosen to extract features from DEAP EEG signals.

A sequence’s Discrete Fourier Transform or inverse Discrete Fourier Transform (DFT) can be determined using FFT. Equation (1) expresses the DFT as follows:

(1) $$x\left[k\right]=\overset{N-1}{\underset{n=0}{\sum }}x\left[n\right]e{\frac{-j2\Pi kn}{N}}^{}$$

Equation (1) calculates the series *x*[*k*] of *N*, a complex exponential number, given another series of data *x*[*n*] of length *N*, according to the formula. In this case, *n* is the domain size. DFT is obtained by multiplying each value of a discrete signal *x*[*n*] by an e-power to a function of *n*. The results acquired for a given *n* should then be added together. The calculated complexity of a signal’s DFT is O(N^2^). FFT is much faster than DFT, as its name implies. Hence, the complexity can be reduced from O(N^2^) to O(NlogN) through FFT.

A total of 14 EEG channels were chosen for this study. Fp1, AF3, F3, F7, FC1, P3, PO3, Fp2, Fz, F4, F8, C4, P4, and PO4 are the electrodes used in the Emotive Epoc+ EEG headset [81] and selected for this study. Five bands were chosen. These include Theta, Alpha, Low Beta, High Beta, and Delta [48]. Their corresponding frequency is shown in Table 3.

**Figure 4:**
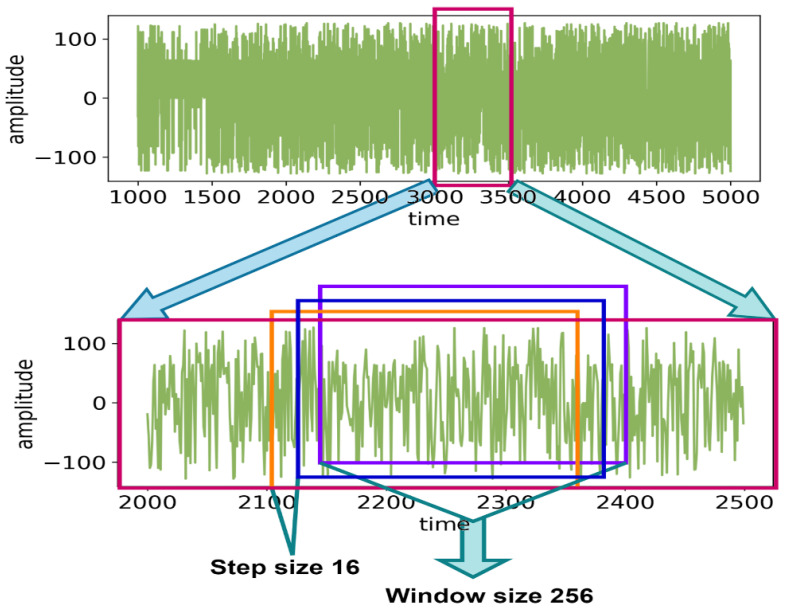
Sliding the window using FFT.

The study explored various window sizes, shown in the experiments and results section. For each of the experiments, the step size is 16, and the sample rate is 128 Hz. FFT incorporates data based on the window size from the raw EEG data. After that, it moves forward by 16 steps, and onward to the end until all of the data is finished. This working procedure and the window size of 256 are shown in Figure 4.

**Figure 5:**
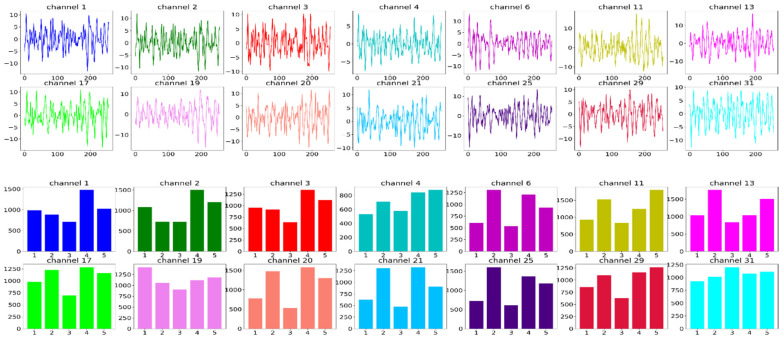

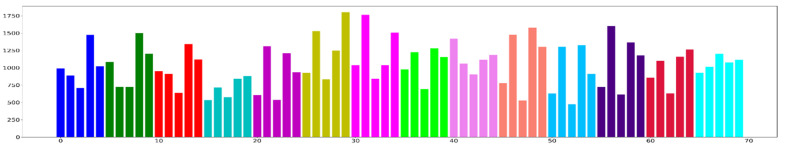
Steps of FFT on selected 14 EEG channels and 5 sub-bands of DEAP dataset. A total of 14 channels [channel no: 1, 2, 3, 4, 6, 11, 13, 17, 19, 20, 21, 25, 29, and 31] are selected and 2 s of EEG data is segmented which consists of 256 floating points. This goes into the FFT function and the FFT is applied on every channel which is shown in the 2nd graph. In this final step, all the channel-wise FFT output is merged together to form a single data point of the data. A single data point has 70 values (14 channels × 5 FFT bands).

In Figure 5, the steps of FFT on each EEG channel are shown. In the first step, all 40 EEG channels’ raw data from the DEAP dataset are loaded for a single participant. In the second step, the selected 14 channels of EEG data are shown together. In the third step, each of the 14 channels of EEG data is plotted. Step four shows how FFT converts each channel signal into a frequency domain by using five bands of power (Theta, Alpha, Low Beta, High Beta, Gamma). The last step represents the frequency domain of all 14 channels together. Here, each channel has five bands, and thus a total of 70 (14 × 5) values are shown.

### 3.4. Data Preparation

Most natural data is not structured in a way suitable for model training. In this study, the EEG signals are represented in tensors, a best practice for representing such data for model training. Both raw and feature extracted data are used for experiments in this paper. For the raw experiments, a 75/25 train–test split is used. All 40 channels are utilized for the raw experiments and the array size for both train and test is shown in Table 4. For this study, two labels (valence, arousal) of the DEAP datasets are used.

For the feature extracted experiments, FFT is applied to each EEG file, and their array size is shown in Table 5. After applying FFT, each EEG data file has 19,520 rows and 70 columns. The 70 columns are a result of selecting 14 channels and 5 sub-bands (14 × 5 = 70) when applying FFT. The output of the FFT is normalized using the StandardScaler() function from the Python sklearn library. Each label EEG file contains 19,520 rows and 4 columns (valence, arousal, dominance, liking). For all 32 participants, the total number of rows is 624,640 (19,520 × 32). For this experiment, only two columns of valence and arousal are used. The information is then prepared to be fed into the CNN model.

**Figure 6:**
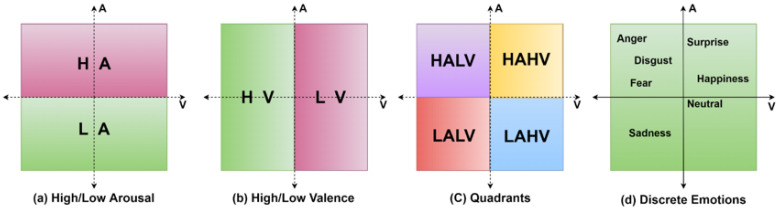
The typical set of emotions recognized on the valence and arousal labels.

The valence and arousal labels are classified into two classes: 1–4.9 in class 0, and 5–9 in class 1. This follows the quadrants in subfigure c from Figure 6. This quadrant subfigure consists of HAHV, HALA, LAHV, and LALV parts where H, L, A, and V denote high, low, arousal, and valence, respectively. The quadrants include emotions shown in subfigure c from Figure 6.

With the use of categorical functions, the arousal and valence labels are categorized. The 2D arrays of the DEAP dataset are converted into 3D using a reshape function because the proposed model requires 3D data as input.

### 3.5. Model Architecture

Deep learning models are composed of an input layer, one or many hidden layers, and an output layer for classification. Moreover, each layer can be customized with different numbers of parameters, kernels, neurons, weights, biases, and activation functions. As a result, the model architecture plays a very important role in achieving good performance. As discussed in the literature review, LSTM [50,51] and Bi-LSTM [46] are frequently used in the DEAP dataset. Thus, an LSTM model and a Bi-LSTM model are selected. However, some studies [48,54] also achieve better results with CNN.

Text and 1D signals are two major applications of 1D Convolutional Neural Networks [82]. CNN has proven to be very effective in different fields of pattern recognition from images [83,84]. Importantly, several studies utilized the pattern recognition power of 1D CNN, including smartphone sensor signal recognition [85,86,87], and ECG signal recognition [88]. Therefore, the current study continues to test its performance with CNN models as well. To recognize emotion from the DEAP dataset, five different 1D CNN models are used, with varying numbers of layers and parameters. A residual connection is used to improve the performance of 1D CNN, except for the one with one convolution and two dense layers. Residual connections give additional ways for data to reach later regions of the neural network by skipping over some layers. To implement these residual connections, the sequential model is converted into a functional model.

## 4. Experiments and Results

In this section, each of the four research questions (RQ) mentioned in the introduction to this paper is answered based on extensive experimentation and analysis. The experiments and results section is broken into five subsections. First, through experimentation, the best deep learning model to classify emotions from raw EEG signals is selected. After selecting the deep learning model, parameter reduction experiments are performed to obtain the optimal model with as few parameters as possible. Next, various experiments, including experimentation with different EEG lengths, are conducted to find the effects of adding feature extraction and complementary features. Lastly, various experiments with different training–testing splits are conducted to identify the model’s performance on alternative training sizes as well.

### 4.1. Deep Learning Model Selection

To answer research question RQ1, this experiment is conducted with multiple deep learning models. As EEG signals are time series data, LSTM and CNN can be effective for high-quality recognition. CNN [52,60], LSTM [50,51,55], and Bi-LSTM [55] are the most common models used with the DEAP dataset. For this reason, the LSTM, Bi-LSTM, and CNN models shown in Table 6 are selected for recognizing emotion from brain signals. With the models established, it becomes possible for us to implement the experimental procedure and identify results.

To find out which one is more effective to recognize emotions, the raw EEG from the DEAP dataset is given as input without any kind of preprocessing or feature extraction. All three models are trained on two-class classifications of the valence and arousal labels from the DEAP dataset. To further verify the claim, these models are also trained on the binary classification of EEG data from the Confused Student dataset [89]. The test accuracy of these models is shown in Table 6.

In the DEAP dataset, the LSTM model achieves an average train accuracy of 87.5% and average test accuracy of 47%. The Bi-LSTM model obtains an average train accuracy of 72.5% and an average test accuracy of 31.5%. The difference between the train and test accuracy of both LSTM and Bi-LSTM is very high. It shows that both of these models have overfitting issues, as they have achieved high train accuracy while achieving low test accuracy. All of the deep learning models’ performances reported in Table 6 are lower, as we are using the raw dataset. The raw EEG signals of the DEAP are highly complex, even for deep learning techniques. The Bi-LSTM model is very low compared to the LSTM and CNN, because Bi-LSTM attempts to learn time series data, using forward and backward passes. For most of the language data, the backward pass makes sense. However, for EEG, the backward scan does not introduce any meaningful information. As a result, the backward scan of EEG data creates a negative impact on EEG learning.

On the other hand, the 1D CNN model achieves an average train accuracy of 72.13% and average test accuracy of 63.9%. The CNN model achieves better test accuracy and handles the overfit issue much better when compared to the LSTM and Bi-LSTM models. To further verify the result, the LSTM, Bi-LSTM, and CNN models with the same number of layers are trained on another EEG dataset called the Confused Student dataset. Similar trends were observed, where the CNN model performed best with 56.65% test accuracy. LSTM and Bi-LSTM achieved 53.14% and 47.26% test accuracy, respectively.

The CNN model is selected for the remaining experiments as it outperforms LSTM and Bi-LSTM models in both EEG datasets.

### 4.2. Parameter Reduction

Further delving into RQ1, this particular experiment is designed to obtain a minimum parameterized model with high recognition accuracy. Because CNN performs best in recognizing emotion from brain signals, as shown in Table 6, it is desired to find the most lightweight CNN model that would perform similarly but without losing the significant performance achieved, as shown in Table 7.

**Figure 7:**
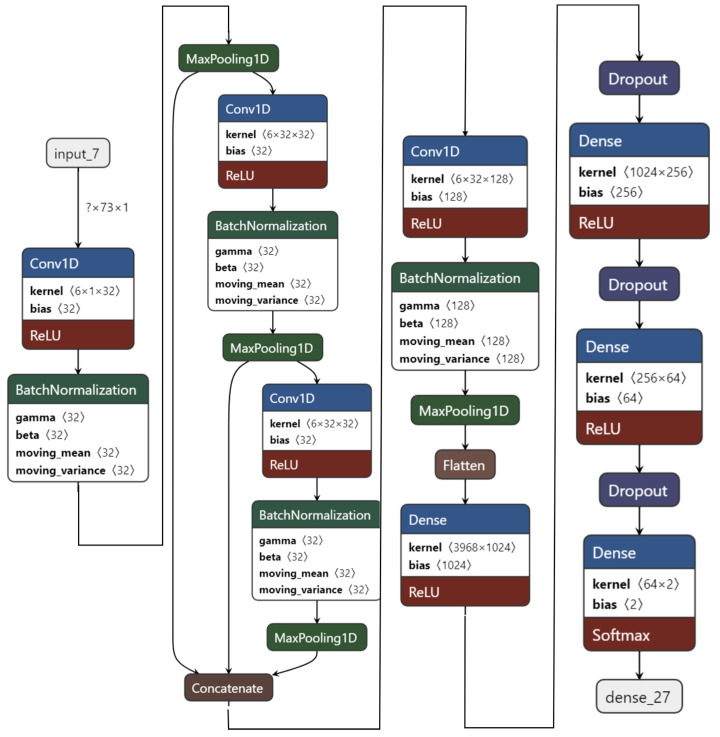
M1 Model: 1D CNN model with 4 convolution and 3 dense layers.

The experiment starts with the 1D CNN as shown in Figure 7. It has four convolution layers and three dense layers. Using FFT as feature extraction, the 1D CNN model is trained on two-class classifications for the valence label.

For this experiment, the ratings of arousal, dominance, and liking are also used as feature extensions to further improve the performance of recognition as all of the four labels of the DEAP dataset are correlated. The 1D CNN model with four convolution layers and three dense layers achieved 99.89% test accuracy on the valence label after 63 epochs. However, this model is not light, since it has over 4,381,410 total parameters.

Thus, one convolution layer and one dense layer are then reduced from this to create a 1D CNN model with three convolution and two dense layers. This model is relatively lighter with a total of 70,130 parameters and it achieves 99.60% test accuracy after 30 epochs. This model is 62 times lighter than the first model in terms of the number of parameters while achieving similar performance.

Thus, one more convolution layer is reduced to achieve 99.44% testing accuracy after thirty epochs, while making the model 73 times lighter than the first model and 1.18 times lighter than the second model.

**Figure 8:**
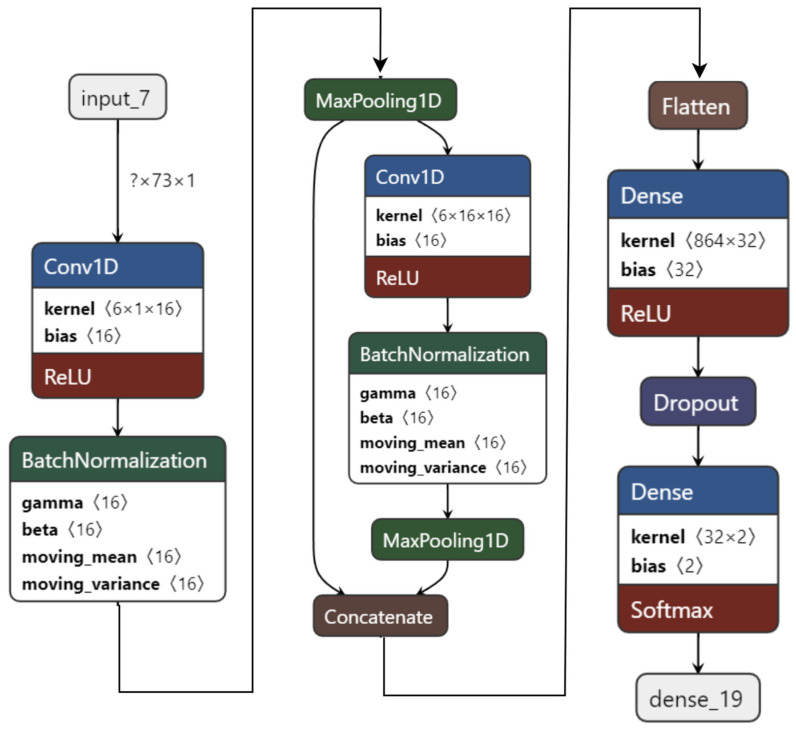
M2 model: 1D CNN model with 2 convolution and 1 dense layers.

As the performance is still good, another dense layer is reduced to create a 1D CNN model with only two convolution layers and one dense layer, as shown in Figure 8. This model has only 29,538 parameters. It is 148 times lighter than the first model, 2.37 times lighter than the second model, and twice as light as the third model. While being light, this 1D CNN model achieves 99.22% testing accuracy on the valence label for two-class classification after 100 epochs.

Next, another CNN model with 1 convolution and 2 dense layers is tested. Though this was the lightest model, with 18,706 parameters, it only achieved 96.92% testing accuracy after 30 epochs. It loses performance greatly as compared to the other models.

**Figure 9:**
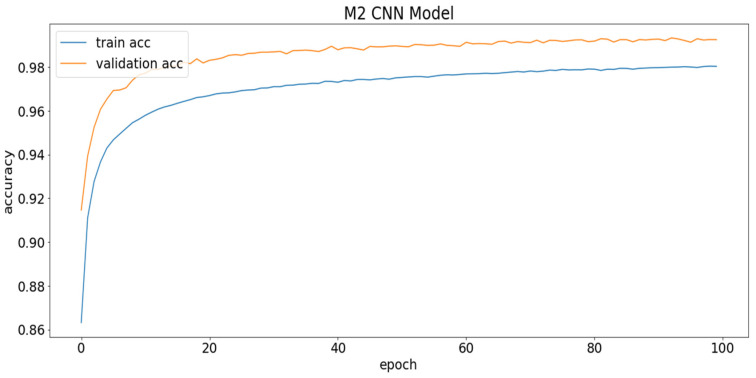
M2 model train vs. validation accuracy graph on valence class.

Among the five models referred to in Table 7, the 1D CNN model with four convolution and three dense layers is the best in terms of testing accuracy. It is selected as the heaviest model (M1) for the remainder of the experiments since it is the heaviest model overall. On the other hand, the 1D CNN model with two convolution layers and one dense layer is selected as the light model (M2) for further experiments, as it is very light in comparison to the others, while not significantly losing performance. The validation accuracy graph of the light model is shown in Figure 9.

To keep the model from overfitting the training data, heavy regularizers are used by adding drop-out. The curves in Figure 9 represent how the model can generalize over the unseen test dataset. Thus, the validation accuracy is better than the training accuracy, which serves as evidence of good generalization over the unseen test data. The hyper-parameters used for these experiments are epochs = 100; batch size = 100; optimizer = Adam; loss function = categorical cross-entropy; activation function = relu (hidden layers) and soft-max (output layer).

### 4.3. Testing with Complementary Features

This experiment answers the research question RQ2. Two CNN models were selected (heavy and light) from the parameter reduction experiment. At this point, CNN models with and without feature extension or complementary features are tested.

In Table 8, the results of the heavy and light 1D CNN model with feature extraction and feature extension (other ratings) are shown. The following are applicable when referring to Table 8: M1 = heavy; M2 = light; FFT = extracted features using FFT; FE = feature extension or complementary features (other scores like dominance, liking, etc.); L = liking, D = dominance; FE-3 = three different scores (dominance, liking, arousal/valence).

While using raw EEG with the M1 model, a 61.56% and 66.25% testing accuracy was achieved for valence and arousal, respectively. However, by using FFT as feature extraction, the M1 model achieved 96.22% and 96.27% testing accuracy on valence and arousal, respectively. This is a very significant improvement over the raw EEG.

To test the effect of feature extension, liking was used as the feature extension on the M1 + FFT + FE-L experiment. This results in 98.79% and 98.6% testing accuracy for valence and arousal, respectively. Adding the dominance rating along with liking further increases the performance shown in the M1 + FFT + FE-L-D experiment. This experiment achieves 99.67% and 99.52% testing accuracy for valence and arousal, respectively.

**Figure 10:**
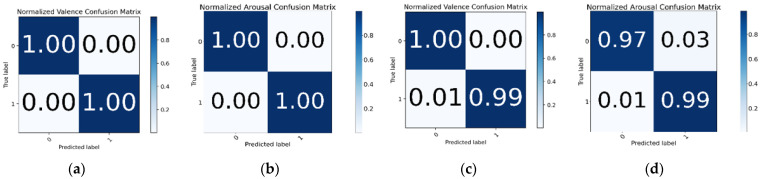
Confusion matrix of valence and arousal label for M1 + FFT + FE-3 and M2 + FFT + FE-3 experiment. (**a**) M1 + FFT + FE-3 experiment on Valence; (**b**) M1 + FFT + FE-3 experiment on Arousal; (**c**) M2 + FFT + FE-3 experiment on Valence; (**d**) M2 + FFT + FE-3 experiment on Arousal.

Three other ratings were added as feature extensions with respect to valence and arousal for improved accuracy. For training valence, these other three ratings (arousal, dominance, and liking) were used as feature extensions and this results in the model achieving 99.89% testing accuracy. For arousal, the M1 model was trained with FFT as feature extraction and the other three ratings (valence, arousal, and liking) as feature extension and this achieved 96.27% testing accuracy. The confusion matrix of valence and arousal for this experiment is shown in Figure 10a,b.

The M2 model achieved 42% and 55% testing accuracy on raw EEG for valence and arousal, respectively. However, the M2 model with FFT as feature extraction achieved 78.61% and 80.22% accuracy for valence and arousal, respectively. This is a marked improvement over only the raw EEG, but as the M2 model is lightweight, its lack of feature extension becomes noticeable in its performance. In the M2 + FFT + FE-L experiment, only “liking” was used as a feature extension rating and the M2 model achieved 91.27% and 89.42% accuracy for valence and arousal, respectively. This demonstrates the importance of feature extension and how it improves the model performance significantly.

Surprisingly, in the M2 + FFT + FE-L-D experiment, using both liking and dominance ratings as a feature extension increases the M2 model’s performance for valence and arousal, which was 97.83% and 96%, respectively. This shows that adding more ratings can lead to an increase in the model’s performance.

In the M2 + FFT + FE-3 experiment, all three ratings were used, achieving 99.22% and 97.80% testing accuracy for valence and arousal, respectively. The confusion matrix for the M2 + FFT + FE-3 experiment on the valence and arousal labels is shown in Figure 10c,d. In Table 8, it is observed that for both M1 and M2 models, the emotion recognition performance increases significantly when applying both feature extraction (FFT) and feature extension (other ratings) together.

Accuracy is a good way to measure a model’s performance. However, accuracy alone without precision can make the model’s actual performance misleading. Accuracy can be calculated using Equation (2). When the classes are severely unbalanced, precision–recall is a helpful indicator of prediction success. Precision in information retrieval refers to how relevant the results are, whereas recall measures the number of outcomes that are relevant. Precision and recall can be calculated using Equations (3) and (4), respectively. The average value of precision and recall is the *F*1 Score. Therefore, both false positives and false negatives are considered while calculating this score. The *F*1 score can be calculated using Equation (5).

(2) $$Accuracy=\frac{TruePositives+TrueNegatives}{TruePositives+FalsePositives+TrueNegatives+FalseNegatives}$$

(3) $$Precision=\frac{TruePositives}{TruePositives+FalsePositives^{\prime }}$$

(4) $$Recall=\frac{TruePositives}{FalseNegative+TruePositives^{\prime }}$$

(5) $$F1\;Score=\frac{2\times Precision\times Recall}{Precision+Recall}$$

In Table 9, the performance report for the M1 and M2 models is shown. The results of Table 9 indicate that the classes are well balanced and, in all cases, the M1 model achieves almost perfect accuracy. The M2 model also performs very well.

In Table 10, the results of previous state-of-the-art works are compared to the proposed M1 and M2 CNN models of this experiment. To obtain these results, FFT-produced frequencies, dominance, and liking ratings were used as features. We report the test accuracy of the valance and arousal classes and the average accuracy of these two labels to compare them with the previous results. Hasan et al. [48] used a 1D CNN model with four convolution layers and three dense layers, which may be considered a heavy model. They also used FFT as a feature extraction technique to achieve 96.63% and 96.17% test accuracy on valence and arousal, respectively, for two-class classification. On the other hand, the proposed light M2 model with only the two convolutions and one dense layer outperforms their accuracy on both valence and arousal labels. The proposed M1 model exceeds their accuracy by an even greater margin.

As shown in Table 10, Yin et al. [47] used a fusion of graph convolution neural network, LSTM, and differential entropy as feature extraction techniques, achieving only 90.45% and 90.60% test accuracy on valence and arousal, respectively. Both of the proposed M1 and M2 models exceed their accuracy by 8–9%. In the following row of Table 10, Ma et al. [60] used multi-modal residual LSTM network without any feature extraction technique and only achieved 92.30% and 92.87% accuracy for valence and arousal, respectively. The proposed M1 and M2 models outperform their model by 7%. Next, Alhalaseh et al. [54] used a CNN model and two feature extraction techniques called entropy and Higuchi’s fractal dimension (HFD). They achieved 95.2% testing accuracy on two-class classification. The proposed light and heavy 1D CNN exceeds their accuracy by 4%. On the next line, Cui et al. [46] achieved 94.02% and 94.86% test accuracy on valence and arousal, respectively, by fusing both CNN and BiLSTM. Their modeling technique is called DE-CNN-BiLSTM and they used differential entropy as a feature extraction technique. Their fusion of CNN and BiLSTM definitely made their model heavy. However, despite using a particularly heavy model, their accuracy falls behind by 5% compared to both of the proposed M1 and M2 models.

**Figure 11:**
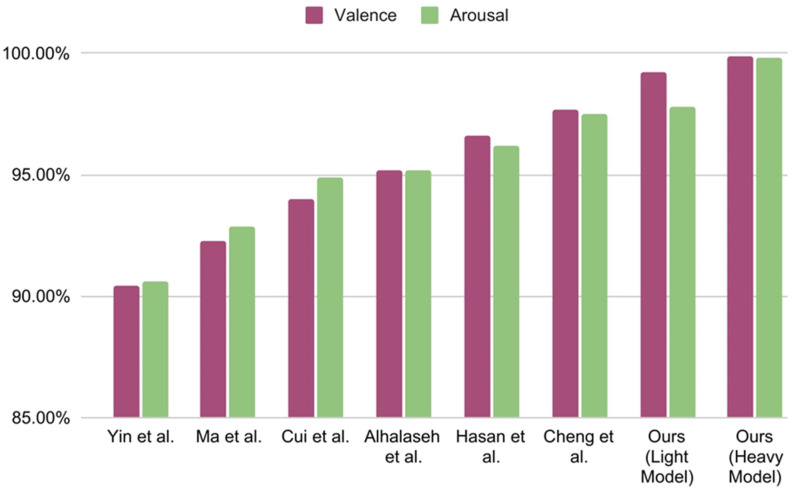
Plotted comparison of state-of-the-art results [46,47,48,54,58,60] from Table 10.

In the current study, both of the proposed CNN models use a residual connection, and the features are extracted using FFT, while the other ratings are used as feature extensions. The result, shown in Table 10 compared with the other aforementioned models, is plotted in Figure 11 for visualizing the result. The proposed 1D CNN model with two convolution layers and one dense layer (the light model) achieves 99.22% and 97.80% test accuracy on valence and arousal, respectively, for binary classification. On the other hand, the 1D CNN model with four convolution layers and three dense layers (heavy model) achieve 99.89% and 99.83% testing accuracy on valence and arousal, respectively. This accuracy is highest even when compared to the other state-of-the-art works shown in Table 10.

### 4.4. The Effective EEG Length

In this section, we explain the solution for research question RQ3. Hasan et al. [48] used an EEG length of 2 s (window size 256) and got 96.18% accuracy for arousal and 96.63% accuracy for valence. In this paper, all the previous experiments have been conducted using a 256-window size, corresponding to a two-second time slice of EEG data. However, to recognize emotion from brain signals in real-time, it becomes necessary to reduce the EEG length as much as possible.

**Figure 12:**
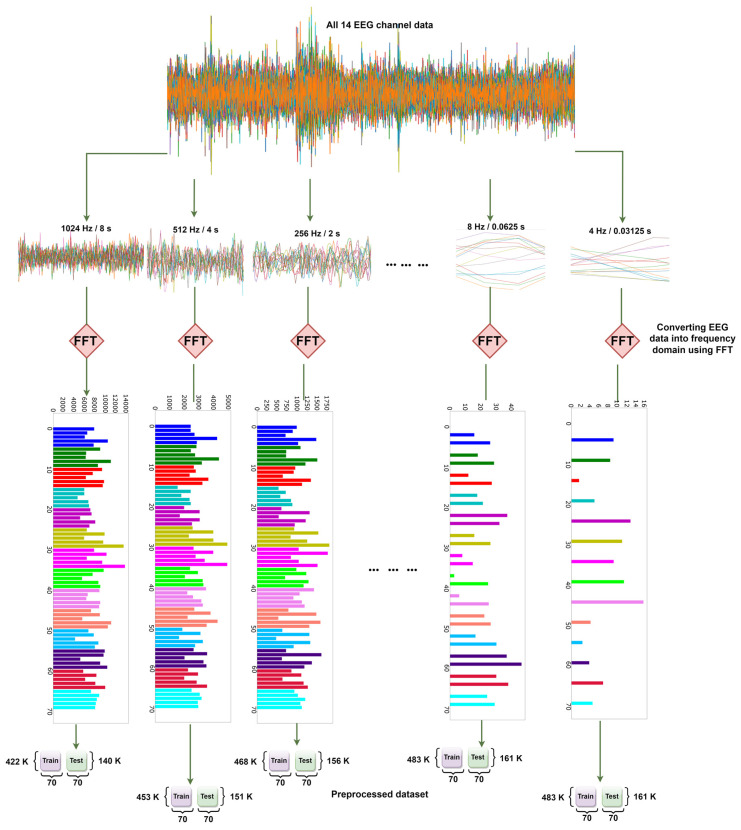
Generating preprocessed data with various window sizes/time slices using FFT.

To experiment with the effects of the window size of FFT on the accuracy, multiple preprocessed files are generated by varying window sizes ranging from 4 to 1024. The process of generating these is shown in Figure 12. First, EEG data with 14 channels are selected, with different window sizes subsequently taken each time. Then, after applying FFT, the preprocessed dataset is ultimately created for that particular window size or time slice.

In Table 11, FFT is used for feature extraction with different window sizes. This is done to test the accuracy of various time slices on DEAP EEG data. As the M1 model achieves better accuracy than the M2 in each of the previous experiments, these experiments are therefore tested using the M2 model (two convolutions + one dense). This is because, if M2 can perform with an accuracy of more than 90%, then M1 will be able to exceed that level of accuracy. Hence, the lighter model is tested, rather than the heavier M1 model, to make the experiment more challenging and reliable. In the DEAP dataset, the sample rate is 128 Hz; thus, using the 128-window size can be considered equal to one second. The window size is changed from 4 to 1024 to conduct the experiments.

**Figure 13:**
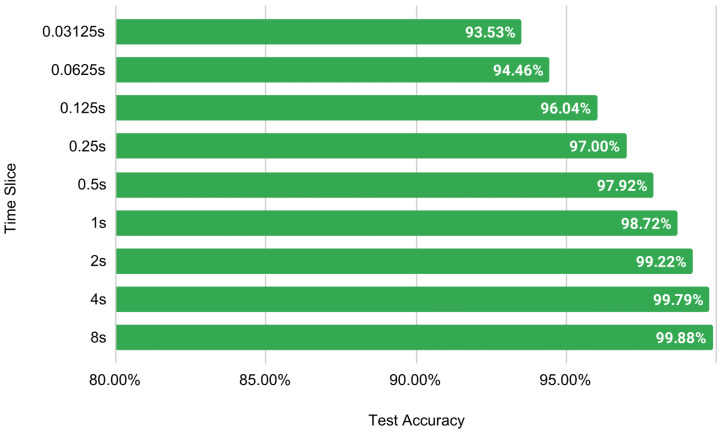
Visualization of the effect of changing time slice on test accuracy using FFT.

To visualize the results clearly, a bar plot is shown in Figure 13. The time slice is directly proportional to the testing accuracy. The light model with two convolutions and one dense layer achieves a respectable 93.53% accuracy with a 0.03-s time slice of EEG data. It shows the possibility of using EEG data to recognize emotions in real-time. For the rest of the experiments, a window size of 256, or two-second time slice, is selected as it provides a balance between lower time slices without losing accuracy significantly.

### 4.5. Training Size Requirement

Finally, solutions for RQ4 are explored in this experiment. Though the proposed models achieved state-of-the-art accuracy, it is possible that using a higher training size may affect the model’s performance. Hence, it is important to understand how effectively the model will perform with different training sizes.

Thus, as shown in Table 12, a different combination of the train–test split set was tested. Each of the experiments was executed in Google Colab using the M2 model for 100 epochs. The required training and testing time is noted for each split. Wall time is the amount of time that a wall clock (or a stopwatch held in the hand) would indicate has passed between the beginning and end of the process. The amount of time spent in user code and the amount of time spent in kernel code are referred to as user-CPU time and system-CPU time, respectively. Among the options available for the train–test split, a 75/25 split attained the best test accuracy of 99.26%.

Google Colab Pro Configuration:

1. CPU = Intel(R) Xeon(R) CPU @ 2.00 GHz
2. GPU = Tesla P100-PCIE
3. GPU Memory = 16 GB
4. GPU Memory Clock = 1.32 GHz

In Table 13, the test accuracy of the M2-CNN model is compared with that of Alhalaseh et al. [54] for different training dataset size splits. Alhalaseh et al. [54] used CNN, K-Nearest Neighbour (KNN), Naïve Bayes (NB), and Decision tree (DT). The common train/test splits between both of the studies were 80/20 and 50/50. In both cases, this paper’s M2-CNN model outperforms the previous study by a sizable margin.

Based on these experiments, all of the stated research questions are restated below and are now answered empirically.

**RQ1:**  *Which deep learning model is more effective for EEG as a form of time series signal recognition?*

Among CNN, LSTM, and Bi-LSTM deep learning models, CNN performs the best on the raw EEG signals. The M1 CNN model contains 4381 K parameters, while the M2 model has only 29.5 K parameters, with similar recognition accuracy. Table 6 and Table 7 represent the corresponding experiment results.

**RQ2:**  *Can feature extraction improve the recognition accuracy of a deep learning model? What is the most effective feature set?*

Features extracted by FFT can boost recognition accuracy of CNN by at least 30% (raw EEG accuracy 66% and FFT feature-based model accuracy 96%). The most effective feature set is a combination of frequency domain features extracted by FFT, with deep features extracted by Conv layers of CNN, and complementary features using additional information such as dominance and liking. Table 8 represents the corresponding experiment results.

**RQ3:**  *What is the minimum EEG signal required for good recognition? Can it be done in real-time?*

Only two seconds of EEG signal are required to attain over 99% accuracy. Even 125 milliseconds of EEG data can achieve more than 96% accuracy for valence. Table 11 and Figure 13 represent the corresponding results.

**RQ4:**  *How much data and time are required for training the model to achieve a reasonable performance?*

Only 50% of the EEG dataset can achieve over 99% accuracy on valence, and only 10% of the dataset can achieve a valence accuracy of 96.84%. The model training time with 100 epochs is approximately 1 h (50% dataset), while the model can predict 176 K data points in under 40 s. Table 12 and Table 13 represent the corresponding results.

## 5. Further Research and Implications

This study holds a myriad of opportunities for further research, in both technical and applied areas. Firstly, the study is performed on subject-dependent emotional recognition from the DEAP dataset. One important subsequent project would be determining if these results could be replicated for subject-independent emotional recognition. For this, instead of the test set being inclusive of each participant’s EEG that is already in the training set, the study would be done with the test set being completely unfamiliar with the subjects’ EEG readings.

This limitation of our study leads to the next potential opportunity for further research, which is the use of additional datasets besides the DEAP. While the DEAP is an outstanding dataset for this kind of research, another limitation of this study is that it is only performed on the DEAP. For further research, therefore, it would be important to study the generalizability of these results with additional datasets, or even completely original participants.

In addition to technological advancements for further research, there are innumerable important applications of sound emotion recognition algorithms. The implications and applications for this work are vast, including as disparate fields as medicine and marketing. It is not only a potential but a current reality that big data is evolving to include emotional data. This may revolutionize how we interact with each other and our environment—an environment that may be able to digitally know our emotions perhaps even before we know them ourselves.

In medicine, emotional recognition can allow for major advances in mental health, as well as tremendous increases in the ability of physicians and nurses alike to utilize empathy and compassion in care. Can mental health counseling ever become equally effective when performed by software, or does a human being need to be involved? Soft robotics promises a future where machines can interact side-by-side with people, safely and humanely collaborating, but how would this be possible without machines having the ability to interpret and respond appropriately to people’s emotions of happiness, sadness, excitement, or fear?

All of these current and future implications offer great benefits to humanity, but also raise ethical concerns. The current paper lays out an increasingly effective method of recognizing emotions from digital data elicited via neural sensors. This paper may be utilized to develop even more effective methods in the future. At some point, emotional recognition will be seamlessly elicited from people’s digital devices everywhere. When that point is reached, data privacy and public policy can become pivotal concerns for everyday people. Often, laws governing people’s private information do not keep up with the advent of new technology, and in this case, people’s most private feelings need consideration on whether and under what circumstances they should be available to others.

## 6. Conclusions

Emotional recognition plays an essential role in human social interaction, mental health, education, and medical practice. It is so crucial to people’s lives that it would be impossible to imagine humanity without considering emotional states such as happiness, sadness, fear, and love. Incredibly, these very emotions, which can be said to make people truly human, can be precisely recognized from brain signals elicited via EEG readings. This paper has worked to elucidate more effectively the translation of EEG brain waves to real human emotions than has been done by previous works.

This study achieves state-of-the-art accuracy using a 1D CNN model with residual connections on the DEAP EEG dataset. FFT is used for frequency domain features and convolution layers when extracting deep features. The study is done on the binary classification of valence and arousal labels of the DEAP dataset. By using complementary features (other ratings of the dataset), the performance is even further enhanced. Two CNN models are proposed: one light model with 7.69% parameters for the dataset size and a second model that is heavy compared to the light model. Among the 40 EEG channels of the DEAP dataset, only 14 channels are used in this study. The light 1D CNN model achieved an average accuracy of 99.22% with only a two-second time slice. With the same time slice, the heavy 1D CNN model achieved an average accuracy of 99.89%. Different EEG lengths were also tested, ranging from 0.03 s to eight seconds, using various window sizes of FFT. Only two seconds of EEG signals are sufficient to achieve good emotion recognition performance. The mentioned accuracy was determined on a 75/25 train–test split. Experiments with an alternative train–test split demonstrated that these proposed models performed exceptionally well even when using only 50% of the dataset.

This study has achieved state-of-the-art accuracy using a 1D CNN model with residual connection on the DEAP EEG dataset. FFT is used for frequency domain features and a convolutional layer for extracting deep features. The study was carried out on the binary classification of valence and arousal labels of the DEAP dataset. The authors also showed that by using complementary features (other ratings of the dataset), the performance can be greatly enhanced. The authors proposed two CNN models, one light with 7.69% parameters for the dataset size and the other one heavy compared to the light model. Among the 40 EEG channels of the DEAP dataset, the authors used only 14 channels for this study. The light 1D CNN model achieved an average accuracy of 99.22% with only a 2 s time slice. With the same time slice, the heavy 1D CNN model achieved an average accuracy of 99.89%. The authors also experimented with different EEG lengths ranging from 0.03 s to 8 s using various window sizes of FFT. The authors showed that only 2 s of EEG signal is enough to achieve good emotion recognition performance. The mentioned accuracy is on a 75/25 train–test split. The authors further experimented with other train–test split and showed that the proposed models can also perform very well using only 50% of the dataset.

## Figures and Tables

**Table 1:** Notable literature for the DEAP on binary classification of valence and arousal labels.

No	Research	Year	Feature Extraction Method	Modeling Technique	Average Test Accuracy
1	Cui et al. [46]	2022	DE	DE-CNN-BiLSTM	94%
2	Yin et al. [47]	2020	DE	GCNN, LSTM	90%
3	Hasan et al. [48]	2021	FFT	CNN	96%
4	Galvão et al. [49]	2021	Hjorth, Spectral Entropy, Wavelet-based features, IMFP and IMFE	KNN, RF	89.8%
5	Anubhav et al. [50]	2020	Common power	LSTM	93.91%
6	Maeng et al. [51]	2020	PSD, Wavelet-based features, FFT	LSTM	93%
7	Gao et al. [52]	2021	PSD, Hjorth, SE, DE	CNN + SVM	77.87%
8	Asghar et al. [53]	2020	DFC	SVM	81.30%
9	Alhalaseh et al. [54]	2020	Entropy and HFD	CNN	95.20%
10	Garg et al. [55]	2019	Wavelet-based features and Stats	LSTM	84%
11	Wang et al. [56]	2019	Time frequency domain	P-GCNN	75.17%
12	Liu et al. [57]	2018	EMD, SBS	KNN, SVM	85%

**Table 2:** Array contents for all 32 participants together in the DEAP dataset.

Array	Shape	Contents
Data	32 × 40 × 40 × 8064	#Subjects × #Video × #Channel × EEG quantized signal
Label	32 × 40 × 4	#Subjects × #Video × Label (Valence, Arousal, Dominance, Liking)

**Table 3:** Frequency range of used sub-bands.

Band	Frequency Range
Theta	4–8 Hz
Alpha	8–12 Hz
Low Beta	12–16 Hz
High Beta	16–25 Hz
Gamma	25–45 Hz

**Table 4:** Array size for train–test split of 75/25 ratio for raw EEG experiments.

Array	Shape	Contents
Train data	32 × 30 × 40 × 8064	#subject × 75% of videos × #Channel × EEG quantized signal
Train label	32 × 30 × 2	#subject × 75% of videos × Label (Valence, Arousal)
Test data	32 × 10 × 40 × 8064	#subject × 25% of videos × #Channel × EEG quantized signal
Test Label	32 × 10 × 2	#subject × 25% of videos × Label (Valence, Arousal)

**Table 5:** Individual array size of data and label arrays after applying FFT using 14 channels and 5 bands.

Array	Shape	Contents
Data	19,520 × 70	#windows × (14 channels × 5 bands)
Label	19,520 × 4	#windows × Label (Valence, Arousal, Dominance, Liking)

**Table 6:** Results summary of two-class classifications of valence and arousal from DEAP dataset and two-class classifications from confused student dataset with LSTM, BiLSTM, and CNN models using raw EEG data.

Model	Architecture	Test Accuracy for DEAP Dataset		Test Accuracy for Confused Student Dataset
		Valence Class	Arousal Class	
LSTM	2 LSTM layers + 2 dense layers	48%	46%	53.14%
Bi-LSTM	2 Bi-LSTM layers + 2 dense layers	35%	28%	47.26%
**CNN**	**4 Conv layers + 3 dense layers**	**61.56%**	**66.25%**	**56.65%**

**Table 7:** Selecting the most optimal 1D CNN model.

#Layers	#Parameters	Parameter–Data Ratio: (Parameters/#Data Size × 100%)	Test Accuracy (Valence Class)
**4 Conv + 3 Dense (M1)**	**4,381,410**	**1140.99**	**99.89%**
3 Conv + 2 Dense	70,130	18.26	99.60%
2 Conv + 2 Dense	59,298	15.44	99.44%
**2 Conv + 1 Dense (M2)**	**29,538**	**7.69**	**99.22%**
1 Conv + 2 Dense	18,706	4.87	96.92%

**Table 8:** Results summary of heavy (M1) and light (M2) 1D CNN models with feature extraction and feature extension.

Experiments	Test Accuracy of Valence Class	Test Accuracy of Arousal Class
M1 + FFT	96.22%	96.27%
M1 + FFT + FE-L	98.79%	98.6%
M1 + FFT + FE-L-D	99.67%	99.52%
**M1 + FFT + FE-3**	**99.89%**	**99.83%**
M2 + FFT	78.61%	80.22%
M2 + FFT + FE-L	91.27%	89.42%
M2 + FFT + FE-L-D	97.83%	96.00%
**M2 + FFT + FE-3**	**99.22%**	**97.80%**

**Table 9:** Performance report for M1 and M2 models with binary classification for 75/25 train–test split.

Model Name	Label	Classes	Precision	Recall	*F*1-Score	Support
M1	Valence	0.00	1.00	1.00	1.00	67,832
		1.00	1.00	1.00	1.00	88,328
	Arousal	0.00	1.00	1.00	1.00	64,172
		1.00	1.00	1.00	1.00	91,988
M2	Valence	0.00	0.99	1.00	0.99	67,832
		1.00	1.00	0.99	0.99	88,328
	Arousal	0.00	0.98	0.97	0.98	64,172
		1.00	0.98	0.99	0.98	91,988

**Table 10:** Comparison of our results with state-of-the-art results on binary classification on DEAP.

Research	Modeling Technique	Feature Extraction	Test Accuracy		
			Valence	Arousal	Average
Hasan et al. [48]	CNN	FFT	96.63%	96.17%	96.4%
Yin et al. [47]	GCNN, LSTM	DE	90.45%	90.60%	90.52%
Ma et al. [60]	MMResLSTM	Raw	92.30%	92.87%	92.58%
Alhalaseh et al. [54]	CNN	SE and HFD	95.2%	95.2%	95.2%
Cui et al. [46]	DE-CNN-BiLSTM	DE	94.02%	94.86%	94.43%
**Ours**	**1D CNN (M2-Light)**	**FFT and FE**	**99.22%**	**97.80%**	**98.51%**
**Ours**	**1D CNN (M1-Heavy)**	**FFT and FE**	**99.89%**	**99.83%**	**99.86%**

**Table 11:** Varying window size with respective time slice using FFT on M2 + FFT + FE-3 for valence.

Window Size	Time Slice	Training Size	Test Size	Test Accuracy
4	0.03125 s	483,840	161,280	93.53%
8	0.0625 s	483,840	161,280	94.46%
16	0.125 s	482,880	160,960	96.04%
32	0.25 s	481,920	160,640	97%
64	0.5 s	480,000	160,000	97.92%
128	1 s	476,160	158,720	98.72%
256	2 s	468,480	156,160	99.22%
512	4 s	453,120	151,040	99.79%
1024	8 s	422,400	140,800	99.88%

**Table 12:** Accuracy with different train/test splits and their effect on time.

Train/Test Split	Sample Size		Train				Sample Size		Test			
			Training Time			Train Accuracy			Testing Time			Test Accuracy
	Class 0	Class 1	User	Sys	Wall		Class 0	Class 1	User	Sys	Wall	
80%/20%	217,077	282,635	1 h 5 min 52 s	9 min 58 s	1 h 4 min	97.86%	54,251	70,677	15.3 s	2.52 s	15.3 s	99.02%
75%/25%	203,496	264,984	47 min 22 s	4 min 38 s	43 min 48 s	98.06%	67,832	88,328	14.2 s	1.66 s	20.8 s	99.26%
50%/50%	135,664	176,656	54 min 40 s	7 min 42 s	52 min 24 s	97.76%	135,664	176,656	38.2 s	6.05 s	37.6 s	99.19%
20%/80%	54,251	70,677	34 min 7 s	3 min 37 s	43 min 22 s	97.39%	217,077	282,635	47 s	4.71 s	44s	98.35%
10%/90%	27,119	35,345	38 min 13 s	6 min 33 s	38 min 23 s	96.34%	244,209	317,967	1 min 8 s	11.4 s	1 min 22 s	96.84%

**Table 13:** Comparison of performance based on required training dataset size.

Paper	Train/Test (%)	Model	Avg. Test Accuracy
Alhalaseh et al. [54]	80/20	CNN	94.93%
		KNN	94.03%
		NB	92.27%
		DT	88.50%
**Our Proposed**	**80/20**	**M2-CNN**	**99.02%**
Alhalaseh et al. [54]	50/50	CNN	94.26%
		KNN	90.45
		NB	91.91
		DT	90.15
**Our Proposed**	**50/50**	**M2-CNN**	**99.19%**
**Our Proposed**	**90/10**	**M2-CNN**	**96.84%**

## Data Availability

Data for this study is available at: https://www.eecs.qmul.ac.uk/mmv/datasets/deap/ (accessed on 25 October 2022).

## Code Availability Statement

https://github.com/PiasTanmoy/M1M2-Deep-Learning-Based-Real-Time-Emotion-Recognition-from-Neural-Activity (accessed on 25 October 2022).
